# Coping With Primary Progressive Aphasia: Factors Predicting Caregiver Psychological Wellbeing and Burden

**DOI:** 10.1111/1460-6984.70095

**Published:** 2025-07-25

**Authors:** Johan Wong, David Foxe, James Carrick, Rebekah M. Ahmed, James R. Burrell, Olivier Piguet

**Affiliations:** ^1^ Brain and Mind Centre The University of Sydney Sydney Australia; ^2^ School of Psychology The University of Sydney Sydney Australia; ^3^ School of Medical Sciences The University of Sydney Sydney Australia; ^4^ Memory and Cognition Clinic, Department of Clinical Neurosciences Royal Prince Alfred Hospital Sydney Australia; ^5^ Concord Clinical School, Medical Education Centre, Concord General Hospital The University of Sydney Sydney Australia

**Keywords:** caregiving, coping, dementia, frontotemporal dementia, primary progressive aphasia

## Abstract

**Background:**

Impacts of dementia syndromes on caregivers are well established, but research specific to Primary Progressive Aphasia (PPA) populations is scant. In particular, little is known about the impacts of non‐language symptoms (e.g., emotion recognition and behavioural disturbance) on caregiver outcomes in PPA.

**Aims:**

The present study sought to investigate the interrelationships between non‐language symptom profiles, caregiver coping behaviours, and caregiver outcomes (psychological wellbeing and burden) among PPA subtypes.

**Methods & Procedures:**

Ninety‐six PPA person‐caregiver dyads (30 with logopenic variant [lvPPA], 26 with non‐fluent variant [nfvPPA], and 40 with semantic variant [svPPA]) and 122 healthy controls were included in this cross‐sectional study. Caregiver outcomes were assessed using the Zarit Burden Interview (ZBI) and the 21‐item depression, anxiety, and stress scale (DASS‐21). The study investigated whether a person with PPA‐focused variables (psychological wellbeing, emotion recognition ability, behavioural disturbance) and caregiver coping style predicted caregiver outcomes. Differential caregiver coping styles were also examined.

**Outcomes & Results:**

Overall, caregivers most commonly used adaptive coping styles (problem‐focused, emotion‐focused). Symptom profiles and use of dysfunctional coping correlated negatively with caregiver psychological wellbeing and positively with burden. Regression models indicated that caregiver psychological wellbeing was most strongly predicted by the use of dysfunctional coping strategies, and caregiver burden was most strongly predicted by reduced emotion recognition and presence of behavioural disturbance symptoms in persons with PPA.

**Conclusions & Implications:**

This study highlights the importance of considering non‐language symptoms in persons diagnosed with PPA and their impact on caregiver outcomes. These findings may inform the development of psychoeducation materials and interventions for PPA caregivers. Further research is needed to identify the predictors of PPA caregiver outcomes with disease progression. Studies utilising qualitative approaches and considering caregiver gain are warranted to understand the experience of PPA caregivers further.

**WHAT THIS PAPER ADDS:**

*What is already known on this subject*

- Impacts on dementia caregivers are well established, including reduced psychological wellbeing and high perceived caregiver burden. However, little is known about the effects of increasingly documented non‐language symptoms (behavioural disturbance, emotion recognition impairment) in persons with Primary Progressive Aphasia (PPA), and whether caregiver coping behaviour influences these outcomes.

*What this paper adds to the existing knowledge*

- This study is the first to investigate the interrelationships between symptom presentation in the person with PPA, caregiver coping behaviours, and caregiver outcomes in PPA subtypes. Our investigations identified the combination of risk factors most strongly predictive of impacted caregiver outcomes, which varied according to PPA subtype. Overall, caregiver burden was most strongly predicted by the person with PPA‐focused variables (emotion recognition, behavioural disturbance), while caregiver psychological wellbeing was most strongly predicted by caregivers’ use of dysfunctional coping.

*What are the potential or actual clinical implications for this work?*

- Our findings inform intervention design, identify targets for psychoeducation, and suggest directions for future research. To best preserve PPA caregivers’ psychological wellbeing, supporting the development of adaptive coping skills appears crucial. Levels of caregiver burden should be monitored especially closely when persons with PPA showcase non‐language symptoms, with protective measures such as respite care implemented pre‐emptively.

## 1 Introduction

Primary Progressive Aphasias (PPAs) are dementia syndromes characterised by early and significant changes in receptive and/or expressive language skills, while other cognitive skills and behaviour are comparatively preserved in the early disease stages (Gorno‐Tempini et al. 2011). Three PPA subtypes are generally recognised, based primarily on distinct language features: semantic (svPPA), non‐fluent (nfvPPA), and logopenic (lvPPA) variants of PPA (Gorno‐Tempini et al. 2011).

svPPA is characterised by a progressive loss of semantic knowledge, typified by anomia (inability to recall everyday object names) and impaired single‐word comprehension, with preserved speech and grammar (Gorno‐Tempini et al. 2011). svPPA is associated with focal atrophy of the lateral and ventral portions of the anterior temporal lobe, most notably in the left hemisphere. nfvPPA is typified by slow and effortful speech, speech sound errors, impaired single‐word repetition, and sometimes agrammatism. Single‐word comprehension and object knowledge generally remain unaffected (Gorno‐Tempini et al. 2011). nfvPPA is associated with progressive atrophy of the left posterior inferior fronto‐insular region, extending into bilateral subcortical areas and the left frontal regions with disease progression. Finally, lvPPA is characterised by deficits in single‐word retrieval in naming and spontaneous speech, impaired repetition of phrases and sentences, and impaired sentence comprehension; single‐word comprehension, grammar, and object knowledge generally remain unaffected (Gorno‐Tempini et al. 2011). lvPPA is associated with unilateral brain atrophy involving the left temporo‐parietal junction area, potentially extending to the medial temporal lobe region with disease progression.

The impact of these language difficulties is far reaching. Indeed, persons with PPA report experiencing significant communication challenges which impact participation and quality of life (Davies et al. 2024; Foxe, Ainkaran et al. 2024).

### 1.1 Non‐Language Symptoms

Mounting evidence indicates the presence of changes beyond language in PPA even in the early stages of the disease, including behavioural disturbance and impaired emotion processing (Hardy et al. 2024). Accurate diagnosis may thus be challenging, as some persons with PPA present with only minor or mixed language symptoms.

Behavioural disturbances have been documented in all PPA syndromes, including reduced motivation, disinhibition and abnormal motor behaviour (Foxe et al. 2022), and difficulty recognising familiar faces (Kumfor et al. 2015). Impaired emotion processing, including the reduced ability to correctly identify others’ emotional states based on cues such as posture and facial expression, has also been documented (Gressie et al. 2024). Evidence shows impaired emotion recognition, especially for negative emotions such as anger and sadness (Gressie et al. 2024).

### 1.2 Caregiver Outcomes

Caregivers of persons living with dementia often experience greater emotional, financial, and physical difficulties compared to caregivers of persons without dementia (Kasper et al. 2015). Studies of dementia caregivers report reduced quality of life (Tulek et al. 2020) and elevated levels of anxiety, depression and stress (Ying et al. 2018; Zwerling et al. 2016). The demands of dementia caregiving are compounded by the care recipient's memory loss, cognitive deficits, impaired functioning in daily activities, and increased dependency—factors which often culminate in transition to supported care facilities (Correa et al. 2013). For PPA caregivers, the younger onset age is likely to exacerbate disruptions to professional, financial and social aspects of life, notwithstanding the unexpectedness of chronic illness at that life stage (Rasmussen et al. 2019). Studies examining PPA caregiver outcomes are scant, but existing findings indicate elevated levels of depression, anxiety and stress (Wong and Wallhagen 2012), emotional detachment and reduced quality of life (Pozzebon et al. 2017), and high perceived burden (Wong et al. 2020).

Caregiver outcomes are crucial to consider. Dementia caregiver burnout is associated with an increased risk of abuse of the care recipient (Yan 2014), and increased caregiver burden is a primary indicator of transition to supported accommodation and nursing home placements, both of which result in a loss of independence and increased health care costs (Afram et al. 2014).

### 1.3 Caregiver Coping

Coping involves affective, behavioural, and cognitive responses to stress, with implications for psychological and physical well‐being (Gottlieb and Wolfe 2002). Research on dementia caregiving generally classifies coping strategies into three main styles: problem‐focused, emotion‐focused and dysfunctional (Li et al. 2012). Problem‐focused coping aims to directly reduce the stressor, employing strategies such as planning and utilising instrumental support. Emotion‐focused coping does not directly modify the stressor; instead, it utilises strategies including emotional support and humour to alter its meaning. Last, dysfunctional coping encompasses strategies such as substance use and venting.

Associations between coping strategies and caregiver outcomes have been previously reported in different populations, for example, in caregivers of older relatives or of persons diagnosed with Alzheimer's disease (Rodríguez‐Pérez 2017). Problem and emotion‐focused approaches are associated with improved wellbeing and reduced burden (Wong and Wallhagen 2012). Conversely, dysfunctional coping is linked to higher levels of caregiver stress, depression, and burden (Li et al. 2012; Lloyd et al. 2019), and increased tendency to explore alternative care arrangements, including residential care (Gallagher et al. 2011). Whether such links between different caregiver coping styles and caregiver outcomes are also present in PPA has not been investigated.

Changes in the person with dementia are similarly associated with caregiver outcomes; reduced caregiver psychological wellbeing is linked to increased behavioural disturbance (Terum et al. 2019), impaired emotion recognition (Kumfor et al. 2014), and low levels of psychological wellbeing (Feast et al. 2016), while increased caregiver burden is linked to behavioural disturbance (Wong et al. 2020). In light of the interdependence in outcomes between caregiver and care recipient, identifying the risk factors most strongly associated with PPA caregiver outcomes is crucial to the development of strategies to improve caregiver outcomes.

### 1.4 Research Aims and Hypotheses

This study aimed to investigate the interrelationships between the non‐language symptoms in persons with PPA, caregiver coping, and caregiver outcomes across PPA subtypes. Our hypotheses were: (1) Impacted caregiver outcomes (reduced psychological wellbeing; increased burden) would be negatively associated with emotion recognition and psychological wellbeing in the person diagnosed with PPA, and positively associated with behavioural disturbance and caregiver use of dysfunctional coping. (2) Behavioural disturbance, emotion recognition ability, psychological wellbeing, and caregiver use of dysfunctional coping would significantly predict caregiver outcomes (psychological wellbeing and burden).

## 2 Materials and Methods

### 2.1 Participants

All participants were recruited from the frontotemporal dementia research clinic at the university between 2008 and 2020, and provided written consent to having their data collected and utilised for research purposes. This study was performed in line with the principles of the Declaration of Helsinki. Approval was granted by the South Eastern Sydney Local Health District Human Research, the University of New South Wales and the University of Sydney Ethics Committees.

All participants who met clinical diagnostic criteria for either svPPA, nfvPPA or lvPPA (Gorno‐Tempini et al. 2011) over the study period were included. Diagnosis was established by consensus among a neurologist, a neuropsychologist and an occupational therapist, based on clinical investigations, cognitive assessment, structural neuroimaging, and caregiver interviews. Participants were only included if all measures were completed within six months of the caregiver's coping assessment, resulting in a pool of 96 PPA person‐caregiver dyads (svPPA = 40; nfvPPA = 26; lvPPA = 30) and 122 healthy controls.

Control participants were selected from a volunteer database. All scored ≥88/100 (established dementia threshold) on the Addenbrooke's Cognitive Examination, revised (ACE‐R; Mioshi et al. 2006) or third version (ACE‐III; Hsieh et al. 2013) and performed within normal limits on a standard neuropsychological test battery. Exclusion criteria for all participants included concurrent psychiatric, neurological, and psychological disorders (e.g. schizophrenia, stroke, traumatic brain injury, major depression) or presence of alcohol or substance abuse. All caregivers identified as the primary caregiver of the person diagnosed with PPA, either living with them or seeing them for at least 1 h/day, 5 days/week.

### 2.2 Caregiver Measures

Caregiver coping style was assessed by either the 60‐item Coping Orientations to Problems Experienced (COPE; Carver et al. 1989) or the abbreviated 28‐item Brief‐COPE (Carver 1997). Respondents indicated their likelihood to employ coping strategies such as acceptance, humour, and substance use, grouped into 15 subscales of 4 items each in the COPE, and 14 subscales of 2 items each in the Brief‐COPE. Item scores range from 0, ‘I usually don't do this at all’, to 4, ‘I usually do this a lot’.

In this study, data were available from both versions of the test. To maximise data usage, subscale scores were converted to percentage scores (based on the maximum possible score for each subscale) to allow for combined analyses. Higher percentage scores indicate a stronger preference to employ a coping strategy. Only the 13 subscales common to both versions were considered. Omitted subscales were ‘self‐blame’ (Brief‐COPE) and ‘suppression’ and ‘restraint’ (COPE).

Subscales were grouped into three broad coping styles as per previously reported methodology (Cooper et al. 2008). **Dysfunctional coping** included the substance abuse, denial, mental disengagement, behavioural disengagement, and venting subscales. **Problem‐focused coping** comprised the active coping, planning, and seeking social support for instrumental reasons subscales. **Emotion‐focused coping** included the humour, acceptance, positive reinterpretation and growth, seeking social support for emotional reasons, and religion subscales. Composite scores for each style were calculated (mean of the constituent subscale percentage scores). Higher composite scores indicate a stronger preference for the coping style.

Two caregiver outcome measures of interest were considered. First, burden was assessed with the 12‐item ZBI (Bédard et al. 2001). Items on the ZBI are rated on a five‐point scale with a maximum score of 48. Scores ≥12 indicate clinically significant levels of burden. Second, psychological wellbeing was assessed with the 21‐item depression, anxiety and stress scale (DASS‐21; Henry and Crawford 2005; Lovibond and Lovibond 1995). Scores on the three subscales were examined individually and summed to give a composite total. Higher scores indicate lower levels of psychological wellbeing. Accepted normative cut‐off scores were used to define normal to mild symptom severity: ≤6 for depression, ≤5 for anxiety, and ≤9 for stress (Henry and Crawford 2005).

All caregiver measures were self‐administered and were completed on the day of the clinic visit or at home within two weeks of the visit. These measures were selected for their ease of administration and are all commonly used in the dementia field.

### 2.3 Person With PPA Measures

These measures were completed at the clinic as part of the standard diagnostic work‐up. Disease severity was assessed by the Frontotemporal Lobar Degeneration‐Modified Clinical Dementia Rating Scale (CDR‐FTLD; Knopman et al. 2008); sum of scores across all domains (Sum of Boxes; CDR‐FTLDSoB) was used to indicate overall functional ability. General cognition was assessed with either the ACE‐R (Mioshi et al. 2006) or the ACE‐III (Hsieh et al. 2013). Where necessary, ACE‐R scores were converted to ACE‐III scores using validated methods (So et al. 2018).

Emotion recognition ability was assessed by the Facial Affect Selection Task (FAST) (Kumfor et al. 2014). Using this task, participants are shown identical faces displaying the six basic emotions and one neutral expression, then asked to select the emotion spoken by the examiner (e.g., ‘Point to the *angry* face’). Forty‐two item sets were presented in total, and all faces were drawn from the NimStim stimulus database (Tottenham et al. 2009). Higher scores represent stronger emotion recognition ability.

Psychological wellbeing was assessed by the self‐reported DASS‐21 (Henry and Crawford 2005; Lovibond and Lovibond 1995). Administration of this measure was carried out together with the clinician, who was able to clarify or rephrase the questions whenever needed, to ensure full comprehension and reliability of the responses by the person with PPA.

Finally, the caregiver‐rated 45‐item Cambridge Behavioural Inventory Revised (CBI‐R; Wear et al. 2008) assessed behavioural disturbance across 10 domains, including eating habits, self‐care, and motivation in the person with PPA. The questions identify frequency as well as severity of changes across these domains. A composite total score was calculated, where higher scores indicate greater behavioural disturbance.

### 2.4 Statistical Analyses

Data were analysed using IBM SPSS Statistics 24.0. Distribution normality of data for demographic, neuropsychological and behavioural variables was first determined using Shapiro–Wilks tests. As many of the variables in the study were not normally distributed, these were normalised before conducting the analyses. Analyses conducted with the dataset transformed for normality yielded near identical findings to the analyses conducted on the original data. Given that parametric tests are fairly robust in the face of violation of normality (for a detailed discussion, see e.g., Norman 2010), we elected to report the analyses conducted with the original data for ease of interpretation.

Demographic, clinical, and neuropsychological test performance variables were compared across groups using univariate Analysis of Variance (ANOVA) tests, followed by Sidak corrected post‐hoc tests. Sex was analysed using a Chi‐square test. Associations between caregiver outcomes and care recipient variables were investigated using Pearson correlational analyses. The Bonferroni correction was used to account for multiple comparisons, setting statistical significance at *p *< 0.008.

Group differences between caregivers’ use of the three coping styles were examined using a repeated measures mixed model ANOVA, followed by Sidak corrected post‐hoc tests. Finally, group differences in caregiver psychological wellbeing and burden were examined using univariate ANOVAs followed by Sidak corrected post‐hoc tests.

Hierarchical regression analyses were conducted for each PPA group to investigate predictors of caregiver psychological wellbeing and burden. Based on previous research on caregiver outcomes, select caregiver (dysfunctional coping style) and person with PPA (emotion recognition, behavioural disturbance, psychological wellbeing) variables were included. The caregiver variable was entered in Model 1. Variables from the person with PPA were added to Model 2 to investigate potential effects on the predictive efficacy of Model 1. Except for correlational analyses, statistical significance for all tests was set at *p *< 0.05.

## 3 Results

### 3.1 Demographic and Cognitive Measures

Demographic and clinical characteristics of the study samples are detailed in Table 1. Participants were matched on demographic variables including sex, age and education. The svPPA group had a significantly longer disease duration than the lvPPA group (*p* < 0.05). No other group differences in disease duration were observed. Dementia severity (CDR‐FTLDSoB) was significantly greater in all PPA groups than in controls, and significantly greater in the svPPA group than in the nfvPPA group (*p* < 0.001).

**TABLE 1:** Demographic and clinical characteristics of persons with primary progressive aphasia and healthy controls.

	Controls	lvPPA	nfvPPA	svPPA	*F*	*p*	Post‐hoc (Sidak corrected)
Number	122	30	26	40	–	–	–
Age (y)	66.0 (6.9)	66.5 (6.8)	68.0 (9.7)	65.6 (6.7)	0.70	0.55	–
Sex (m:f)	59:63	14:16	12:14	19:21	0.06^a^	0.99	–
Education (y)	13.5 (2.9)	12.6 (2.9)	13.0 (3.0)	12.5 (2.9)	1.73	0.16	–
Disease duration	–	4.7 (2.5)	5.3 (2.7)	6.5 (2.7)	4.20	0.02	lvPPA < svPPA
CDR‐FTLD SoB	0.3 (0.6)	4.7 (2.9)	3.7 (2.5)	5.3 (3.1)	55.75	<0.001	All PPA groups < Controls; svPPA < nfvPPA
ACE‐III Total	94.3 (3.6)	60.8 (16.2)	81.8 (7.4)	64.5 (14.4)	167.66	<0.001	All PPA groups < Controls; lvPPA, svPPA < nfvPPA
FAST	38.2 (3.1)	30.7 (5.9)	34.9 (4.7)	30.0 (6.4)	46.29	<0.001	All PPA groups < Controls; lvPPA, svPPA < nfvPPA

*Note*: Values are mean scores with standard deviations in brackets where appropriate. Number of missing values: Disease duration: 3 nfvPPA; CDR‐FTLD SoB: 1 lvPPA, 1 nfvPPA, 52 Controls; ACE‐III: 1 nfvPPA.

Abbreviations: ACE‐III: Addenbrooke's Cognitive Examination—Third edition; CDR‐FTLD SoB: clinical dementia rating – frontotemporal lobar degeneration sum of boxes; FAST, facial affect selection task; lvPPA, logopenic variant; nfvPPA, non‐fluent variant; PPA, Primary Progressive Aphasia; svPPA, semantic variant.

^a^Chi‐Square test.

All PPA groups showed significantly impaired general cognitive ability (ACE‐III Total) and FAST compared to controls. The nfvPPA group significantly outperformed the lvPPA and svPPA groups on both tasks (both *p* values < 0.001). The persons with PPA were also assessed on cognitive measures of language, visuospatial ability, and working memory (Appendix A). Profiles of performance on these tasks were consistent with those previously reported in the literature and are thus not reported here.

### 3.2 Clinical Measures: Person With PPA

Results are detailed in Table 2. The svPPA group exhibited significantly higher levels of behavioural disturbance (CBI‐R) than the lvPPA and nfvPPA groups (both *p* values < 0.001). Compared to controls, both nfvPPA and svPPA groups reported lower levels of psychological wellbeing (DASS‐21) (both *p* < 0.001). Additionally, the svPPA group reported significantly lower psychological wellbeing than the lvPPA group (*p* < 0.001). The majority (>70%) of all PPA groups scored in the normal to mild range of clinical severity ratings for all subscales of depression, anxiety and stress.

**TABLE 2:** Clinical characteristics of persons with primary progressive aphasia, caregivers, and healthy controls.

	Controls	lvPPA	nfvPPA	svPPA	*F*	*p*	Post‐hoc (Sidak corrected)
CBI‐R	–	16.9 (10.8)	10.8 (9.5)	24.3 (14.6)	9.91	<0.001	svPPA > lvPPA, nfvPPA
PwD Total DASS‐21	4.1 (5.3)	8.2 (6.8)	13.7 (11.1)	14.9 (13.5)	21.87	<0.001	nfvPPA, svPPA > Controls; svPPA > lvPPA
NM Depression	–	80.0	73.1	70.0			
NM Anxiety	–	93.3	92.3	77.5			
NM Stress	–	96.7	84.6	80.0			
Caregiver Total DASS‐21	4.1 (5.3)	8.2 (7.4)	7.1 (10.0)	9.0 (6.1)	7.81	<0.001	lvPPA, svPPA > Controls
NM Depression	–	90.0	88.5	87.5			
NM Anxiety	–	90.0	96.2	92.5			
NM Stress	–	86.7	88.5	95.0			
ZBI	–	12.0 (7.2)	8.7 (7.0)	15.3 (9.8)	4.94	0.01	nfvPPA < svPPA
High Burden	–	50.0	26.9	60.0			

*Note*: Unless specified, values are mean scores with standard deviations in brackets where appropriate.

Abbreviations: CBI‐R, Cambridge Behaviour Inventory Revised; DASS‐21, 21‐item depression, anxiety and stress scale; High Burden, percentage of scores meeting threshold for clinical significance; lvPPA, logopenic variant; nfvPPA, non‐fluent variant; NM Depression/Anxiety/Stress, percentage of scores with clinical severity ratings of normal or mild; PPA, primary progressive aphasia; PwD, person with dementia; svPPA, semantic variant; ZBI, Zarit burden interview.

### 3.3 Caregiver Measures

**FIGURE 1:**
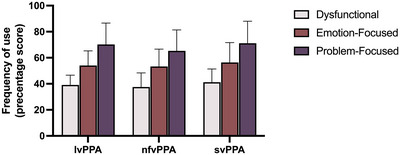
Use of coping styles in caregivers of persons with primary progressive aphasia. Bar graph depicts frequency of use (percentage scores) of problem‐focused, emotion‐focused, and dysfunctional coping strategies by caregivers, clustered by Primary Progressive Aphasia (PPA) group (logopenic [lvPPA], nonfluent [nfvPPA], and semantic [svPPA] variants). Error bars represent standard deviations. No significant between‐group differences were observed for any of the three coping styles (*p* < 0.5). Within each group, problem‐focused coping was used significantly more often than emotion‐focused coping, and both these styles were used significantly more than dysfunctional coping (*p* < 0.5 for all).

PPA caregivers’ use of coping styles is illustrated in Figure 1. No significant between‐group differences were observed for any of the three coping styles. (Appendix B1–B7). In other words, PPA caregivers adopted almost identical approaches to coping; problem‐focused coping was used the most, and dysfunctional coping was used the least. Within each group, problem‐focused coping was utilised significantly more often than emotion‐focused coping, and both these styles were used more than dysfunctional coping (Appendix B1–B7).

Reported level of burden (ZBI) differed significantly across clinical groups (Table 2). svPPA caregivers experienced greater burden than nfvPPA caregivers (*p* < 0.05). Sixty per cent (24/40) of svPPA caregivers reported experiencing high burden (ZBI score ≥ 12/48), compared with 50% (15/30) in lvPPA and only 27% (7/26) in nfvPPA. Finally, self‐reported general psychological wellbeing (DASS‐21), was significantly lower in lvPPA and svPPA caregivers than in controls (*p* < 0.001) (Table 2). No other group differences were observed on this measure. Notably, most caregivers (>85%) scored in the normal to mild range of clinical severity ratings for depression, anxiety, and stress.

### 3.4 Correlation Analyses

Correlational analyses between symptoms in the person with PPA, caregiver coping style, and caregiver outcomes were carried out with all groups combined (Table 3). Caregiver outcomes (psychological wellbeing; burden) were associated with caregiver use of dysfunctional coping and presence of behavioural disturbance (positive correlations), and with difficulties with emotion recognition and psychological wellbeing of the person with PPA (negative correlations). In other words, the presence of non‐language symptoms such as impaired psychological wellbeing, difficulty recognising emotion, and behavioural disturbance, and caregivers’ use of dysfunctional coping strategies, were all associated with impacted caregiver outcomes. No significant correlations were observed with emotion‐focused or problem‐focused coping styles.

**TABLE 3:** Pearson correlations between caregiver use of dysfunctional coping and person with dementia and caregiver clinical variables.

	Dysfunctional coping	FAST	CBI‐R	PwD total DASS‐21	Caregiver total DASS‐21	ZBI
Dysfunctional coping	–					
FAST	−0.26^*^	–				
CBI‐R	0.16	−0.27^**^	–			
PwD total DASS‐21	0.36^***^	−0.20	0.32^***^	–		
Caregiver total DASS‐21	0.52^***^	−0.28^**^	0.33^***^	0.37^***^	–	
ZBI	0.48^***^	−0.47^***^	0.61^***^	0.42^***^	0.60^***^	–

*Note*: *n* = 96 for all correlations.

Abbreviations: CBI‐R, Cambridge Behaviour Inventory Revised; DASS‐21, depression, anxiety and stress scale; FAST, facial affect selection task; PwD, person with dementia; ZBI, Zarit Burden Interview.

* Correlation is significant at the 0.05 level (2‐tailed).

** Correlation is significant at the 0.01 level (2‐tailed).

*** Correlation is significant at the 0.001 level (2‐tailed).

### 3.5 Regression Analyses—Psychological Wellbeing

For the overall PPA sample, both models were significant (Table 4). Model 1 explained 28% of variance. Inclusion of the person with PPA variables in Model 2 explained an additional 8% of variance, with behavioural disturbance the only additional significant predictor. Caregiver use of dysfunctional coping remained a significant predictor. For lvPPA, neither model was significant, although behavioural disturbance in Model 2 was a significant predictor. For nfvPPA, both models were significant. Model 1 explained 60% of variance; inclusion of the person with PPA variables in Model 2 accounted for an additional 12% of variance, but caregiver use of dysfunctional coping remained the only significant predictor. For svPPA, both models were significant. Model 1 explained 24% of variance; inclusion of the person with PPA variables in Model 2 accounted for an additional 13% of variance, with caregiver use of dysfunctional coping being the only significant predictor.

**TABLE 4:** Predictors of psychological wellbeing in caregivers of persons with primary progressive aphasia.

Group	Model	*R^2^*	*F*	*F* Change	Predictor	*B*
PPA Overall	1	0.28^*^	35.61^*^	35.61^*^	Caregiver use of dysfunctional coping	0.52^*^
	2	0.36^*^	12.96^*^	4.20^*^	Caregiver use of dysfunctional coping	0.42^*^
					PwD Psychological Wellbeing	0.15
					Emotion recognition	−0.10
					Behavioural disturbance	0.19^*^
lvPPA	1	0.03	0.88	0.88	Caregiver use of dysfunctional coping	0.18
	2	0.26	2.16	2.53	Caregiver use of dysfunctional coping	0.26
					PwD psychological wellbeing	−0.17
					Emotion recognition	0.00
					Behavioural disturbance	0.48^*^
nfvPPA	1	0.60^*^	35.40^*^	35.40^*^	Caregiver use of dysfunctional coping	0.77^*^
	2	0.72^*^	13.47^*^	3.08	Caregiver use of dysfunctional coping	0.48^*^
					PwD psychological wellbeing	0.37
					Emotion recognition	−0.10
					Behavioural disturbance	0.09
svPPA	1	0.24^*^	12.22^*^	12.22^*^	Caregiver use of dysfunctional coping	0.49^*^
	2	0.37^*^	5.11^*^	2.32	Caregiver use of dysfunctional coping	0.40^*^
					PwD psychological wellbeing	0.13
					Emotion recognition	−0.26
					Behavioural disturbance	0.10

Abbreviation: lvPPA, logopenic variant; nfvPPA, non‐fluent variant; PPA, primary progressive aphasia; PwD, person with dementia; svPPA, semantic variant.

* Indicates statistical significance at *p* < 0.05.

### 3.6 Regression Analyses—Caregiver Burden

For the overall PPA sample, both models were significant (Table 5). Model 1 explained 23% of variance; inclusion of the person with PPA variables in Model 2 explained an additional 36% of variance, where behavioural disturbance and emotion recognition were additional significant predictors. Caregiver use of dysfunctional coping remained a significant predictor. For lvPPA, only Model 2 was significant, explaining 52% of the variance. Only emotion recognition and behavioural disturbance were significant predictors. For nfvPPA, both models were significant. Model 1 explained 15% of variance; inclusion of the person with PPA variables in Model 2 explained an additional 47% of variance, where psychological wellbeing and behavioural disturbance were significant predictors. Caregiver use of dysfunctional coping, however, was no longer a significant predictor in the model. For svPPA, both models were significant. Model 1 explained 39% of variance; inclusion of the person with PPA variables in Model 2 explained an additional 26% of variance, where emotion recognition and behavioural disturbance were additional significant predictors. Caregiver use of dysfunctional coping remained a significant predictor.

**TABLE 5:** Predictors of burden in caregivers of persons with primary progressive aphasia.

Group	Model	*R^2^*	*F*	*F* Change	Predictor	*B*
PPA Overall	1	0.23^*^	28.61^*^	28.61^*^	Caregiver use of dysfunctional coping	0.48^*^
	2	0.59^*^	33.27^*^	26.92^*^	Caregiver use of dysfunctional coping	0.30^*^
					PwD psychological wellbeing	0.12
					Emotion recognition	−0.25^*^
					Behavioural disturbance	0.46^*^
lvPPA	1	0.04	1.11	1.11	Caregiver use of dysfunctional coping	0.20
	2	0.52^*^	6.69^*^	8.26^*^	Caregiver use of dysfunctional coping	0.19
					PwD psychological wellbeing	0.05
					Emotion recognition	−0.33^*^
					Behavioural disturbance	0.59^*^
nfvPPA	1	0.15^*^	4.30^*^	4.30^*^	Caregiver use of dysfunctional coping	0.39^*^
	2	0.62^*^	8.37^*^	8.41^*^	Caregiver use of dysfunctional coping	−0.05
					PwD psychological wellbeing	0.47^*^
					Emotion recognition	−0.18
					Behavioural disturbance	0.39^*^
svPPA	1	0.39^*^	23.74^*^	23.74^*^	Caregiver use of dysfunctional coping	0.62^*^
	2	0.65^*^	16.06^*^	8.70^*^	Caregiver use of dysfunctional coping	0.49^*^
					PwD psychological wellbeing	0.12
					Emotion recognition	−0.25^*^
					Behavioural disturbance	0.34^*^

Abbreviation: lvPPA, logopenic variant; nfvPPA, non‐fluent variant; PPA, primary progressive aphasia; PwD, person with dementia; svPPA, semantic variant.

* Indicates statistical significance at *p* < 0.05.

## 4 Discussion

This study is the first to investigate the interrelationships between non‐language symptoms in persons with PPA, caregiver coping behaviours, and caregiver outcomes in PPA subtypes. Our investigations identified the variables that influence caregiver outcomes—variables which vary according to PPA subtype. In doing so, this study advances our understanding of how non‐language symptoms and caregiver coping style impact caregivers’ psychological wellbeing and burden. These findings will inform intervention design, identify targets for psychoeducation and suggest directions for future research.

Compared to other PPA variants, persons with svPPA showed greater behavioural disturbance, while those with nfvPPA exhibited more preserved emotion recognition ability, in line with previous findings (e.g., Hardy et al. 2024). Importantly, our investigations supported the predicted associations between symptoms exhibited by persons with PPA, caregiver use of dysfunctional coping, and caregiver outcomes, lending support for our first hypothesis. Regardless of PPA variant, impaired emotion recognition, behavioural disturbance, reduced care recipient psychological wellbeing, and caregiver use of dysfunctional coping were all strongly associated with reduced caregiver psychological wellbeing and increased burden. The findings of this research highlight the important influence that non‐language symptoms in persons with PPA, as well as caregiver coping style, can have on caregiver wellbeing and burden.

Notably, clinically significant challenges to caregiver psychological wellbeing were mostly absent. This finding contrasts with the existing literature and the well‐documented challenges to psychological wellbeing in caregivers of persons with dementia (Collins and Kishita 2020; Kaddour and Kishita 2020). This difference may be explained by the fact that most persons with PPA in this sample had low dementia severity ratings according to published guidelines (O'Bryant et al. 2008). Disease progression in persons with PPA can escalate rapidly, however, and it is plausible that changes in caregiver outcomes may follow a similarly steep trajectory over time. Caregivers may also be impacted in domains not considered here, such as physiological health and quality of life.

Our second hypothesis was partially supported. When we investigated the variables predicting caregiver outcomes in the overall PPA sample, behavioural disturbance and caregiver use of dysfunctional coping predicted both caregiver psychological wellbeing and burden, while emotion recognition predicted only caregiver burden. In contrast, psychological wellbeing of the person with PPA did not significantly predict either caregiver outcome. Importantly, the predictor models varied depending on the PPA subtype. For caregiver psychological wellbeing, caregiver use of dysfunctional coping was the main predictor in nfvPPA and svPPA, but of little importance for lvPPA, where behavioural disturbance played a key role instead. For caregiver burden, behavioural disturbance was a central predictor across all groups. Additionally, emotion recognition was relevant in lvPPA and svPPA but not in nfvPPA, where psychological wellbeing of the person with PPA was imperative instead. Once factors relating to the person with PPA were accounted for, caregiver use of dysfunctional coping was a key determinant of burden only in svPPA.

Considering the overall pattern of predictive associations, it appears that caregivers’ psychological wellbeing relates closely to their coping behaviour. In contrast, caregiver burden is more closely related to the symptoms exhibited by the person with PPA, especially behavioural disturbance and emotion recognition ability. These findings provide important evidence of differential determinants of caregiver outcomes across PPA diagnostic groups, suggesting the need for tailored screening and intervention efforts. Psychoeducation targeted at the largely overlooked non‐language symptoms in PPA is clearly needed, given their roles in exacerbating caregiver burden. Promisingly, caregivers have found psychoeducation interventions helpful to enhance their knowledge about PPA and caregiving skills, with the added benefit of feeling connected to and supported by other caregivers (Schaffer and Henry 2022). Interventions to support caregivers in seeking adaptive alternatives to dysfunctional coping are another avenue worthy of further investigation.

A secondary focus of this study was to examine differential symptom presentation across PPA groups within one sample, and corresponding caregiver coping behaviours. Overall, group symptom profiles were characteristic of each PPA variant as reported in the literature, with a variable combination of impaired general cognition and emotion recognition, alongside behavioural disturbance (Kumfor et al. 2015). While symptom profiles varied across persons diagnosed with PPA, all caregiver groups showed the same general pattern of coping approach, whereby problem‐focused coping was most utilised and dysfunctional coping least utilised.

This propensity towards adaptive coping styles may be explained by the sample characteristics. Participants’ engagement with the clinic granted access to tailored information and support from specialised healthcare professionals—resources that are still lacking in the general population. Indeed, disease‐specific information and psychosocial support have been cited by PPA caregivers as their most important needs (Diehl‐Schmid et al. 2013). Caregivers’ voluntary involvement with the clinic also suggests high support‐seeking motivation, which is associated with greater problem‐focused coping and lesser dysfunctional coping (Van Den Wijngaart et al. 2007).

Arguably, the findings must be interpreted with caution. Firstly, cross‐sectional data and the lack of pre‐disease baseline measures of caregiver psychological wellbeing restrict possible interpretations. Variables preexisting and unrelated to the caregiving situation may also modulate the impact of the situation on the caregiver. These include early‐life events, professional changes, and size and involvement of the social network. Secondly, the characteristics of the sample may limit the generalisability of findings to other caregiver populations. Differences in cultural and economic environments (e.g., collectivist vs. individualist societies; high vs. low socio‐economic status; native vs. migrant populations) are all likely to colour the caregiving experience. Thirdly, interpretations are limited by the quantitative nature of the measures used. Despite their scores not meeting clinical cut‐offs, respondents may well still face challenges and have significant support needs that could be elucidated using qualitative measures. Finally, three subscales of the coping measures were omitted from analyses. Although unlikely to alter the observed pattern of findings, consideration is nonetheless required.

## 5 Conclusions

While strongly associated with and predictive of negative caregiver outcomes overall, variables related to the person diagnosed with PPA (non‐language symptoms, psychological wellbeing) and the caregiver's use of dysfunctional coping appear differentially important across PPA diagnostic groups. Further investigation into the predictive value of these variables on caregiver outcomes is needed, particularly in more diverse caregiver populations. Future research may also consider longitudinal designs to investigate caregiver adjustment to disease progression, explore protective factors related to caregiver gain, and investigate predictors of caregiver use of dysfunctional coping.

## Ethics Statement

This study was performed in line with the principles of the Declaration of Helsinki. Approval was granted by the South Eastern Sydney Local Health District Human Research, the University of New South Wales and the University of Sydney Ethics Committees.

## Consent

Informed consent was obtained from all individual participants included in the study. Participants provided informed consent regarding publishing their data.

## Conflicts of Interest

The authors declare no conflicts of interest.

## Supporting information




**Supporting file**: jlcd70095‐sup‐0001‐SuppMat.pdf

## Data Availability

The data generated during, or analysed as part of the current study, are available from the corresponding author on reasonable request.
