# Supplementary material for: Coping With Primary Progressive Aphasia: Factors Predicting Caregiver Psychological Wellbeing and Burden
Source: Int J Lang Commun Disord. 2025 Jul 25;60(5):e70095. doi: 10.1111/1460-6984.70095 (PMC12291616; doi:10.1111/1460-6984.70095)
Supplement: Supplementary file 1 — Supporting file: jlcd70095‐sup‐0001‐SuppMat.pdf [file JLCD-60-0-s001.pdf]

APPENDIX A: Language, Visuospatial Ability, and Working Memory Performance

	Controls	lvPPA	nfvPPA	svPPA	*F*	*p*	Post-hoc (Sidak corrected)
Number	122	30	26	40	-	-	-
Age (y)	66.0 (6.9)	66.5 (6.8)	68.0 (9.7)	65.6 (6.7)	0.70	.55	-
Sex (m:f)	59:63	14:16	12:14	19:21	.06a	.99	-
Education (y)	13.5 (2.9)	12.6 (2.9)	13.0 (3.0)	12.5 (2.9)	1.73	.162	-
Disease Duration	-	4.7 (2.5)	5.3 (2.7)	6.5 (2.7)	4.20	.018	lvPPA < svPPA
CDR-FTLD SoB (24)	0.3 (0.6)	4.7 (2.9)	3.7 (2.5)	5.3 (3.1)	55.75	< .001	All patient groups > Controls; svPPA > nfvPPA
ACE-III Total (100)	94.3 (3.6)	60.8 (16.2)	81.8 (7.4)	64.5 (14.4)	167.66	< .001	All patient groups < Controls; lvPPA, svPPA < nfvPPA
**Non-Verbal Tests**							
RCFT Copy (36)	32.3 (3.2)	24.9 (8.6)	28.3 (6.0)	31.3 (4.8)	19.74	< .001	lvPPA, nfvPPA < Controls; lvPPA < svPPA
RCFT 3 min (36)	17.5 (5.5)	9.3 (8.1)	15.5 (6.0)	12.2 (6.9)	16.88	< .001	lvPPA, svPPA < Controls; lvPPA < nfvPPA
Digit Span Forward	7.1 (1.2)	4.6 (1.6)	5.5 (1.4)	6.3 (1.4)	33.30	< .001	All patient groups < Controls; lvPPA < svPPA
Digit Span Backward	5.5 (1.3)	2.9 (1.2)	3.8 (1.1)	4.7 (1.2)	39.01	< .001	All patient groups < Controls; lvPPA, nfvPPA < svPPA; lvPPA < nfvPPA
EST	38.2 (3.1)	30.7 (5.9)	34.9 (4.7)	30.0 (6.4)	46.29	< .001	All patient groups < Controls; lvPPA, svPPA < nfvPPA
Patient DASS-21	4.1 (5.3)	8.2 (6.8)	13.7 (11.1)	14.9 (13.5)	21.87	< .001	nfvPPA, svPPA > Controls; svPPA > lvPPA
CBI-R	-	16.9 (10.8)	10.8 (9.5)	24.3 (14.6)	9.91	< .001	svPPA > lvPPA, nfvPPA
**Verbal Tests**							
SYDBAT Naming	26.8 (2.1)	14.7 (7.2)	22.9 (5.2)	8.8 (6.0)	184.75	< .001	All patient groups < Controls; svPPA < lvPPA, nfvPPA; lvPPA < nfvPPA
SYDBAT Repetition	29.9 (0.4)	25.7 (4.9)	21.2 (9.9)	29.0 (1.7)	38.08	< .001	lvPPA, nfvPPA < Controls, svPPA; nfvPPA < lvPPA
SYDBAT Comprehension	29.2 (1.2)	26.0 (3.1)	28.7 (1.5)	19.6 (6.5)	93.26	< .001	lvPPA, svPPA < Controls, nfvPPA; svPPA < lvPPA
SYDBAT Semantic	28.1 (1.5)	25.3 (3.7)	27.3 (2.8)	18.8 (5.6)	88.05	< .001	lvPPA, svPPA < Controls; svPPA < lvPPA, nfvPPA

Note: Values are mean scores with standard deviations in brackets where appropriate.
PPA = Primary Progressive Aphasia; lvPPA = logopenic variant; nfvPPA = nonfluent variant; svPPA = semantic variant.
Measures: CDR-FTLD SoB: Frontotemporal Lobar Degeneration-Modified Clinical Dementia Rating Scale Sums of Boxes; ACE-III: Addenbrooke's Cognitive Examination - Third edition; EST: Emotion Selection Task; RCFT: Rey Complex Figure Test; Digit Span: Wechsler Memory Scale-Third edition; DASS-21: 21-item Depression, Anxiety, and Stress Scale; CBI-R: Cambridge Behaviour Inventory Revised; SYDBAT: The Sydney Language Battery. Number of missing values: Disease duration 3 nfvPPA; CDR-FTLD SoB: 1 lvPPA, 1 nfvPPA, 52 Controls; ACE-III: 1 nfvPPA; RCF Copy: 3 lvPPA, 1 nfvPPA, 1 Control; RCF 3min: 3 lvPPA, 1 nfvPPA, 1 Control. Digit Span Forward: 1 lvPPA, 2 nfvPPA, 1 Control; Digit Span Back: 1 lvPPA, 2 nfvPPA, 1 Control; SYDBAT Comprehension: 2 svPPA, 5 Controls; SYDBAT Semantic: 2 svPPA, 6 Controls; SYDBAT Repetition: 1 lvPPA, 1 svPPA, 7 Controls; SYDBAT Naming: 3 svPPA, 4 Controls.
[a] Chi-Square test.

APPENDIX B: Caregivers' Use of Coping Styles – Supplementary Data

Supplementary Table B.1 – Summary of Post Hoc Tests of Between Group Differences in Use of Coping Styles

	**Post hoc test (Sidak corrected)**
**Dysfunctional Coping**	lvPPA = nfvPPA = svPPA
**Emotion-Focused Coping**	lvPPA = nfvPPA = svPPA
**Problem-Focused Coping**	lvPPA = nfvPPA = svPPA

Supplementary Table B.2 – Summary of Post Hoc Tests of Within Group Differences in Use of Coping Styles

	**Post hoc test (Sidak corrected)**
**lvPPA**	Problem-Focused > Emotion-Focused > Dysfunctional
**nfvPPA**	Problem-Focused > Emotion-Focused > Dysfunctional
**svPPA**	Problem-Focused > Emotion-Focused > Dysfunctional

Supplementary Table B.3 – Descriptive Data

Diagnosis	Coping	Mean	Std. Error	95% Confidence Interval	
				Lower Bound	Upper Bound
lvPPA	Problem-Focused Coping	70.19	3.03	64.18	76.20
	Emotion-Focused Coping	54.00	2.48	49.07	58.94
	Dysfunctional Coping	39.13	1.76	35.63	42.62
nfvPPA	Problem-Focused Coping	65.30	3.25	58.85	71.76
	Emotion-Focused Coping	53.35	2.67	48.05	58.65
	Dysfunctional Coping	37.45	1.89	33.70	41.21
svPPA	Problem-Focused Coping	71.15	2.62	65.94	76.35
	Emotion-Focused Coping	56.39	2.15	52.12	60.66
	Dysfunctional Coping	41.23	1.53	38.20	44.26

Supplementary Table B.4 – Pairwise Comparisons (Between Groups)

Coping	(I) Diagnosis	(J) Diagnosis	Mean Difference (I-J)	Std. Error	Sig.[a]	95% Confidence Interval for Difference[a]	
						Lower Bound	Upper Bound
Problem-Focused Coping	lvPPA	nfvPPA	4.88	4.44	0.62	-5.92	15.68
		svPPA	-0.96	4.00	0.99	-10.69	8.78
	nfvPPA	lvPPA	-4.88	4.44	0.62	-15.68	5.92
		svPPA	-5.84	4.18	0.42	-15.99	4.31

	svPPA	lvPPA	0.96	4.00	0.99	-8.78	10.69
		nfvPPA	5.84	4.18	0.42	-4.31	15.99
Emotion-Focused Coping	lvPPA	nfvPPA	0.66	3.65	1.00	-8.21	9.52
		svPPA	-2.39	3.29	0.85	-10.38	5.60
	nfvPPA	lvPPA	-0.66	3.65	1.00	-9.52	8.21
		svPPA	-3.04	3.43	0.76	-11.38	5.29
	svPPA	lvPPA	2.39	3.29	0.85	-5.60	10.38
		nfvPPA	3.04	3.43	0.76	-5.29	11.38
Dysfunctional Coping	lvPPA	nfvPPA	1.67	2.58	0.89	-4.61	7.96
		svPPA	-2.10	2.33	0.75	-7.77	3.56
	nfvPPA	lvPPA	-1.67	2.58	0.89	-7.96	4.61
		svPPA	-3.78	2.43	0.33	-9.68	2.13
	svPPA	lvPPA	2.10	2.33	0.75	-3.56	7.77
		nfvPPA	3.78	2.43	0.33	-2.13	9.68

Based on estimated marginal means

a. Adjustment for multiple comparisons: Sidak.

Supplementary Table B.5 – Pairwise Comparisons (Within Groups)

Diagnosis	(I) Coping	(J) Coping	Mean Difference (I-J)	Std. Error	Sig.[b]	95% Confidence Interval for Difference[b]	
						Lower Bound	Upper Bound
lvPPA	Problem-Focused Coping	Emotion-Focused Coping	16.186*	2.47	0.00	10.17	22.20
		Dysfunctional Coping	31.064*	3.24	0.00	23.19	38.94
	Emotion-Focused Coping	Problem-Focused Coping	-16.186*	2.47	0.00	-22.20	-10.17
		Dysfunctional Coping	14.878*	2.54	0.00	8.69	21.07
	Dysfunctional Coping	Problem-Focused Coping	-31.064*	3.24	0.00	-38.94	-23.19
		Emotion-Focused Coping	-14.878*	2.54	0.00	-21.07	-8.69
nfvPPA	Problem-Focused Coping	Emotion-Focused Coping	11.956*	2.66	0.00	5.50	18.42
		Dysfunctional Coping	27.853*	3.48	0.00	19.40	36.31
	Emotion-Focused Coping	Problem-Focused Coping	-11.956*	2.66	0.00	-18.42	-5.50
		Dysfunctional Coping	15.896*	2.73	0.00	9.25	22.54
	Dysfunctional Coping	Problem-Focused Coping	-27.853*	3.48	0.00	-36.31	-19.40
		Emotion-Focused Coping	-15.896*	2.73	0.00	-22.54	-9.25
svPPA	Problem-Focused Coping	Emotion-Focused Coping	14.753*	2.14	0.00	9.54	19.96
		Dysfunctional Coping	29.918*	2.80	0.00	23.10	36.74
	Emotion-Focused Coping	Problem-Focused Coping	-14.753*	2.14	0.00	-19.96	-9.54
		Dysfunctional Coping	15.165*	2.20	0.00	9.81	20.52

Dysfunctional Coping	Problem-Focused Coping	-29.918*	2.80	0.00	-36.74	-23.10
	Emotion-Focused Coping	-15.165*	2.20	0.00	-20.52	-9.81

Based on estimated marginal means

*. The mean difference is significant at the .05 level.

b. Adjustment for multiple comparisons: Sidak.

Supplementary Table B.6 – Univariate Tests

Coping		Sum of Squares	df	Mean Square	F	Sig.
Problem-Focused Coping	Contrast	574.94	2.00	287.47	1.05	0.36
	Error	25553.44	93.00	274.77		
Emotion-Focused Coping	Contrast	175.25	2.00	87.62	0.47	0.62
	Error	17212.49	93.00	185.08		
Dysfunctional Coping	Contrast	232.43	2.00	116.21	1.25	0.29
	Error	8652.51	93.00	93.04		

Each F tests the simple effects of Diagnosis within each level combination of the other effects shown. These tests are based on the linearly independent pairwise comparisons among the estimated marginal means.

Supplementary Table B.7 – Multivariate Tests

Diagnosis		Value	F	Hypothesis df	Error df	Sig.
lvPPA	Pillai's trace	0.498	45.711[a]	2.00	92.00	0.00
	Wilks' lambda	0.502	45.711[a]	2.00	92.00	0.00
	Hotelling's trace	0.994	45.711[a]	2.00	92.00	0.00
	Roy's largest root	0.994	45.711[a]	2.00	92.00	0.00
nfvPPA	Pillai's trace	0.410	31.979[a]	2.00	92.00	0.00
	Wilks' lambda	0.590	31.979[a]	2.00	92.00	0.00
	Hotelling's trace	0.695	31.979[a]	2.00	92.00	0.00
	Roy's largest root	0.695	31.979[a]	2.00	92.00	0.00
svPPA	Pillai's trace	0.550	56.320[a]	2.00	92.00	0.00
	Wilks' lambda	0.450	56.320[a]	2.00	92.00	0.00
	Hotelling's trace	1.224	56.320[a]	2.00	92.00	0.00
	Roy's largest root	1.224	56.320[a]	2.00	92.00	0.00

Each F tests the multivariate simple effects of Coping within each level combination of the other effects shown. These tests are based on the linearly independent pairwise comparisons among the estimated marginal means.

a. Exact statistic
